# What Interventional Cardiologists Are Still Leaving to the Surgeons?

**DOI:** 10.3389/fped.2016.00059

**Published:** 2016-06-13

**Authors:** Worakan Promphan, Shakeel A. Qureshi

**Affiliations:** ^1^Queen Sirikit National Institute of Child Health, Bangkok, Thailand; ^2^Evelina London Children’s Hospital, London, UK

**Keywords:** catheter intervention, surgical therapy, congenital heart disease, interventional cardiologist, collaboration

## Abstract

Nowadays, development of new technologies is still ongoing with the ultimate goal of maximizing treatment outcomes with less invasiveness and reduced procedural risk. This review is intended to update on when interventionalists need surgical support in common or emerging problems in congenital heart disease.

## Evolution of Technology for Catheter Intervention Makes the Impossibles Possible

During the last 60 years, medical engineering technology has helped to make important advances in transcatheter treatment of congenital and structural heart disease. Catheter-based therapy in congenital heart disease (CHD) became a reality in 1953 with the first description of pulmonary valvoplasty by Rubio-Alvarez et al. (1). Since then, balloon dilation has proved its efficacy (2, 3) and has become treatment of choice for pulmonary valve stenosis. The next paradigm shift of transcatheter CHD therapy was the novel approach of balloon atrial septostomy (BAS) for cyanotic patients with transposition of the great arteries (TGA) in 1966 by Rashkind and Miller (4). Later in the late 1960s, the first percutaneous occlusion of patent ductus arteriosus (PDA) using Ivalon (polyvinyl alcohol) plug was reported (5). In the ensuing 10 years, transcatheter closure of atrial septal defect (ASD) became a reality with a specially designed Dacron-covered stainless steel umbrella device, reported by King & Mills (6). Thereafter, the 1980s was the era of stainless steel material for endovascular stents and occlusion devices (7–9). In the early 1990s, laser and radiofrequency technologies became major contributors of balloon-assisted valvoplasty in pulmonary valve atresia (10). In the late 1990s, novel design of shape-memory devices made from a nickel and titanium (nitinol) metal alloy completely changed the way physicians treat common CHDs (11–13). Nitinol technology has also been used in self-expandable stent platforms for endovascular stents and percutaneous bioprosthesis valves (14, 15).

## Atrial Septostomy

Percutaneous creation or enlargement of the interatrial communication is the earliest collaborations between interventionalist and surgeon in the history of CHD treatment. Over 50 years, BAS has been stabilizing hypercyanotic transposition of TGA babies prior to corrective surgical repair. However, from recent publications, 43–62% of the cases cannot discontinue prostaglandin E1 after the successful BAS (16–18). Apart from enhancing the oxygenated–deoxygenated blood mixture, BAS remains indicate to augment cardiac output in patients with the right or left heart obstructions (e.g., tricuspid atresia, pulmonary valve atresia associated with RV coronary dependent circulation, mitral atresia, aortic atresia). The complications after BAS are unlikely to occur nowadays. These include balloon rupture, failure in balloon deflation, rupture of the atrial appendage, injury of cardiac structures, and transitory rhythm disturbances (19). Although various additional techniques have been developed over the years for creation or enlargement of the septum in complex anatomy (e.g., blade atrial septostomy, static balloon dilation, radiofrequency perforation or transeptal puncture, and stent implantation of the interatrial septum), the goal of these methods is a bridge to definite surgical treatment.

The majority of common CHDs are now treatable percuteneously. However, surgery is still an option for many of these common CHDs and surgeons are still needed to treat some of these defects.

## Patent Ductus Arteriosus Closure

**Figure 1:**
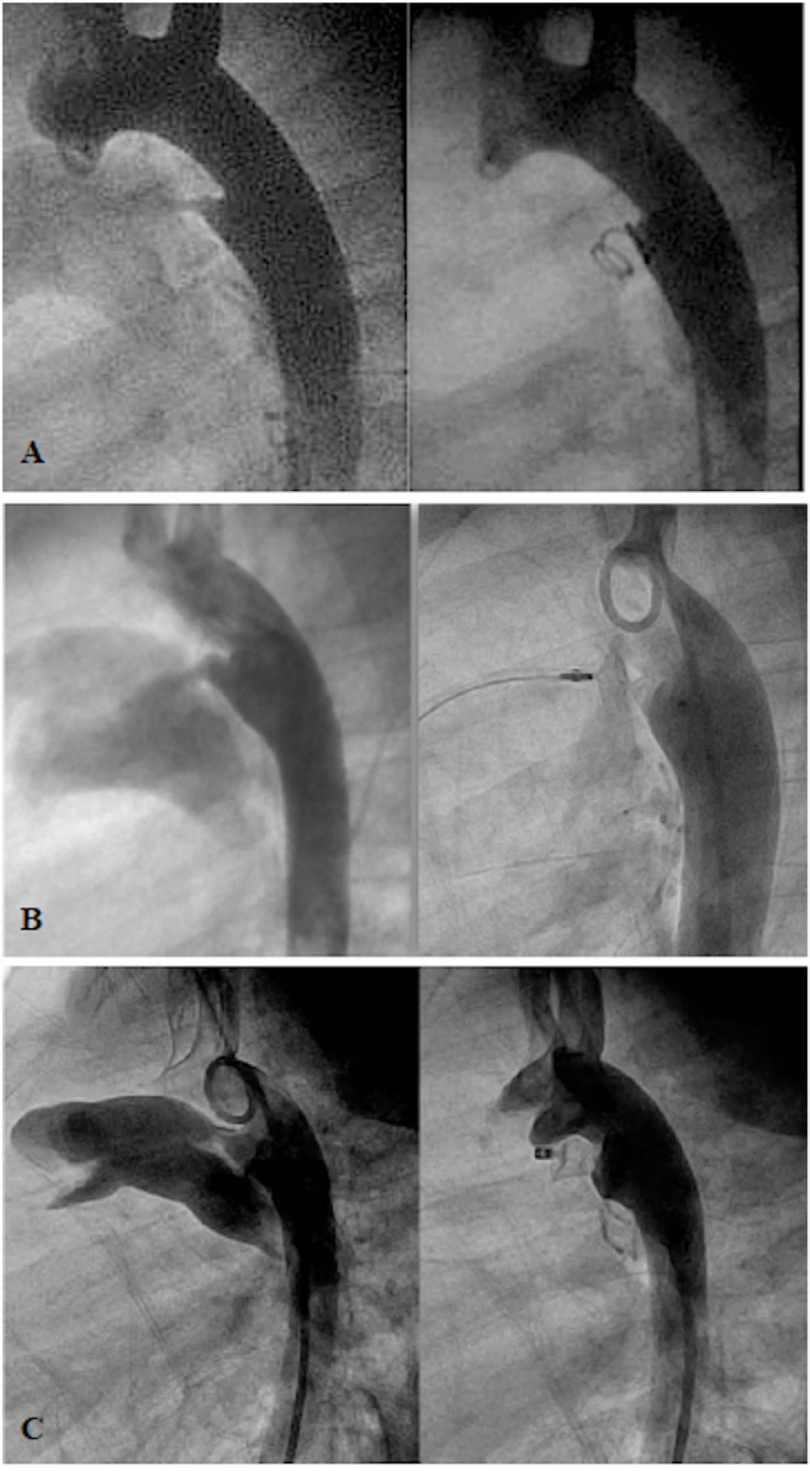
**Angiogram in lateral projection of PDA closed by coil (A), Amplatzer Duct Occluder II (ADOII) (B), and Occlutech Duct Occluder (C)**.

Patent ductus arteriosus is one of the most common CHDs in all ages. Since the first report of transcatheter closure 50 years ago, currently, transcatheter PDA closure has become a widely accepted procedure worldwide with comparable results to surgical therapy (20, 21). Major adverse events of transcatheter PDA occlusion (i.e., device embolization, hemolysis, and obstruction of the left pulmonary artery/aortic isthmus) can occur with incidence of 0.6–83.3% depending upon the selected device (18, 19). It is indicated for a symptomatic moderate to large PDA with left-to-right shunt or previously known occurrence of endocarditis. However, for a small or silent PDA, transcatheter PDA closure may be considered in some countries and avoided in others (22). A variety of devices are available for closing PDAs (23, 24). These have their own advantages and disadvantages. In general, for a small PDA of <2 mm at its narrowest point, closure can be achieved with coils or the Amplatzer Duct Occluder II (ADOII; St Jude Medical, MN, USA) (Figures 1A,B). For a large PDA, the disk devices are preferred (Figure 1C). With custom-made disk devices, a duct of 16-mm diameter or more can be occluded successfully nowadays with a lower risk (25). In addition, with reduction of the delivery system from 18Fr (in 1967) to 3–4 Fr, the feasibility of transcatheter PDA closure is considerably improved, even in newborns (26, 27), and is associated with a shorter recovery time than surgical ligation (28). However, percutaneous PDA closure in neonates and infants is an emerging field with some limitations of available devices and instruments. It seems to have relatively higher major adverse events than in older children (29). Therefore, the procedure has to be balanced with the experience of the operators and the capabilities of the unit, as surgical PDA ligation remains a standard and safe procedure in small babies (30). Further development of a robust device and delivery system that fits to a unique morphology of the duct in prematurity (31) will make transcatheter PDA closure become treatment of choice for symptomatic preterm and/or low-bodyweight infants in the near future.

## Atrial Septal Defect Closure

**Figure 2:**
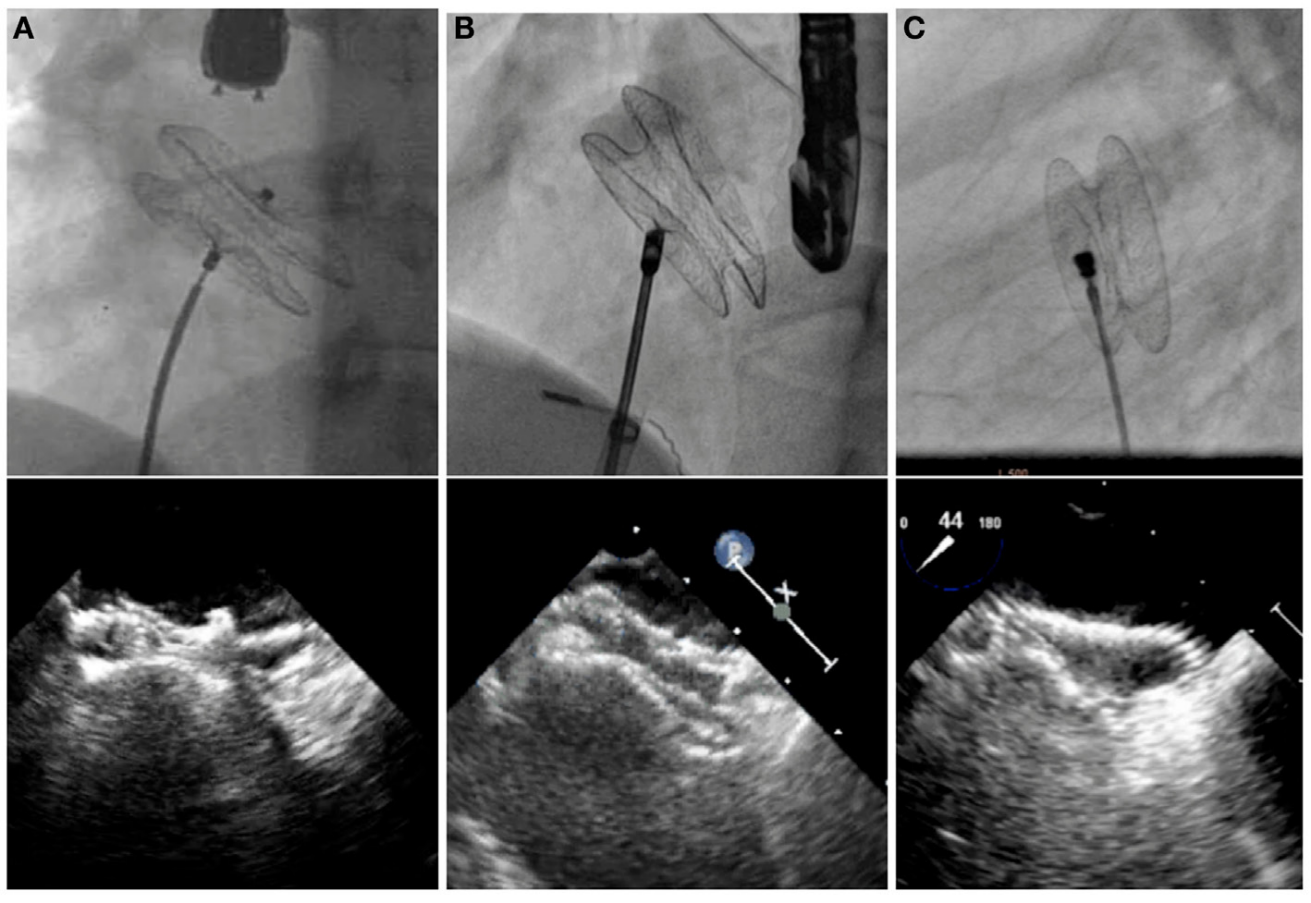
**Fluoroscopic and echocardiographic pictures of various ASD devices**. Amplatzer Septal Occluder **(A)**, Occlutech Figulla Flex II Septal Occluder **(B)**, and CeraFlex ASD Occluder **(C)**.

Double-disk devices are now becoming universal for percutaneous closure of secundum ASDs (Figure 2). In common practice, patients with the body weight more than 15 kg who have hemodynamic significant secundum ASD and suitable anatomical features are indicated for percutaneous ASD closure (22). Transcatheter closure has shown comparable outcome to surgery in pediatric and adult patients, with low complication rates, short anesthetic times, and short hospitalization (32–34). However, once a complication occurs, it sometimes leads to urgent surgical treatment, which is then associated with a higher morbidity and mortality than elective surgical ASD closure (35). Complications after device implantation include device embolization, cardiac perforation, thrombo-embolic events, cardiac arrhythmia, or significant residual shunt. Although the risk of long-term complications after device implantation is very low, several case reports and literature review have shown that these particular problems, such as erosions, may occur 8 years after the initial device closure (36, 37). Therefore, patient selection and comprehensive short- and long-term follow-up are necessary for all transcatheter ASD closure patients. There are some defects with deficient rims in more than one area or very large ASDs, which still require surgical treatment.

## Ventricular Septal Defect Closure

**Figure 3:**
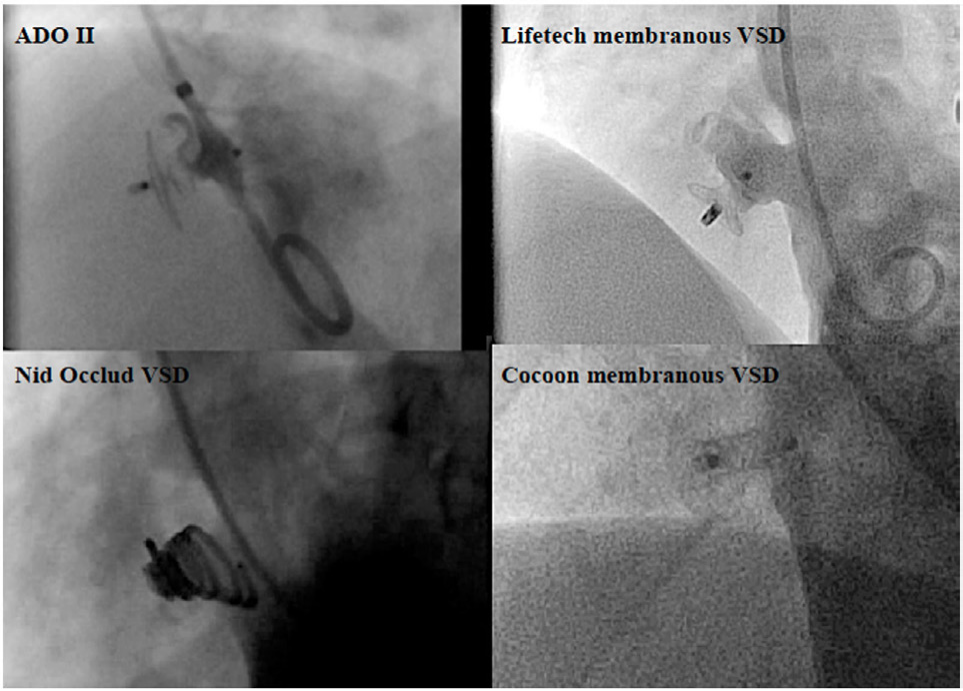
**Angiographic pictures of the occlusion devices for perimembranous ventricular septal defect (pmVSD)**.

Surgery has been the standard treatment of ventricular septal defects (VSDs) for decades. It has been shown to be effective and safe (38). However, with continuous development of novel occlusion devices, transcatheter VSD closure has become an effective alternative treatment modality in appropriately selected patients. The limitation of percutaneous VSD closure relates to the complexity of the defect and the proximity of the defect to adjacent structures, such as aortic, pulmonary, and tricuspid valves. In addition, those VSDs that are close to the membranous area, where the perforating branch of the conduction system lies underneath, closure with a high radial and compression force device may lead to the serious complication of permanent high-grade atrio-ventricular block. During the early phase of transcatheter treatment for perimembranous (pm) VSDs, the eccentric Amplatzer membranous VSD occluder (St Jude Medical, MN, USA) was thought to be effective (39). However, the incidence of post-procedural complete heart block (6–6.5%) was higher than surgery (40, 41). Currently, transcatheter perimembranous ventricular septal defect (pmVSD) closure has not been approved in the US and many countries in Europe. However, in the East, a variety of available devices, which are matched to the different defect morphology and exert less pressure onto the ventricular septum (Figure 3), transcatheter pmVSD closure is considered to be acceptable efficacy, with low complication rates, especially complete heart block (incidence of 0–3%) (41–44). Recently, periventricular mini-thoracotomy VSD closure has emerged as a surgical alternative in China (45, 46). In the special circumstance of muscular or apical VSDs in infants or small children, working in close collaboration with surgeons, an interventional hybrid approach has become an attractive modality (22). Post myocardial infarction VSDs, where surgery may have been ruled out because of unacceptable high risk or in cases of postoperative residual shunt, percutaneous closure may be used (22).

Most cyanotic CHDs usually require surgery. Catheter intervention is a complementary approach to fulfill the optimal treatments in the following circumstances.

## PDA Stenting in Duct-Dependent Pulmonary Circulation Defects

**Figure 4:**
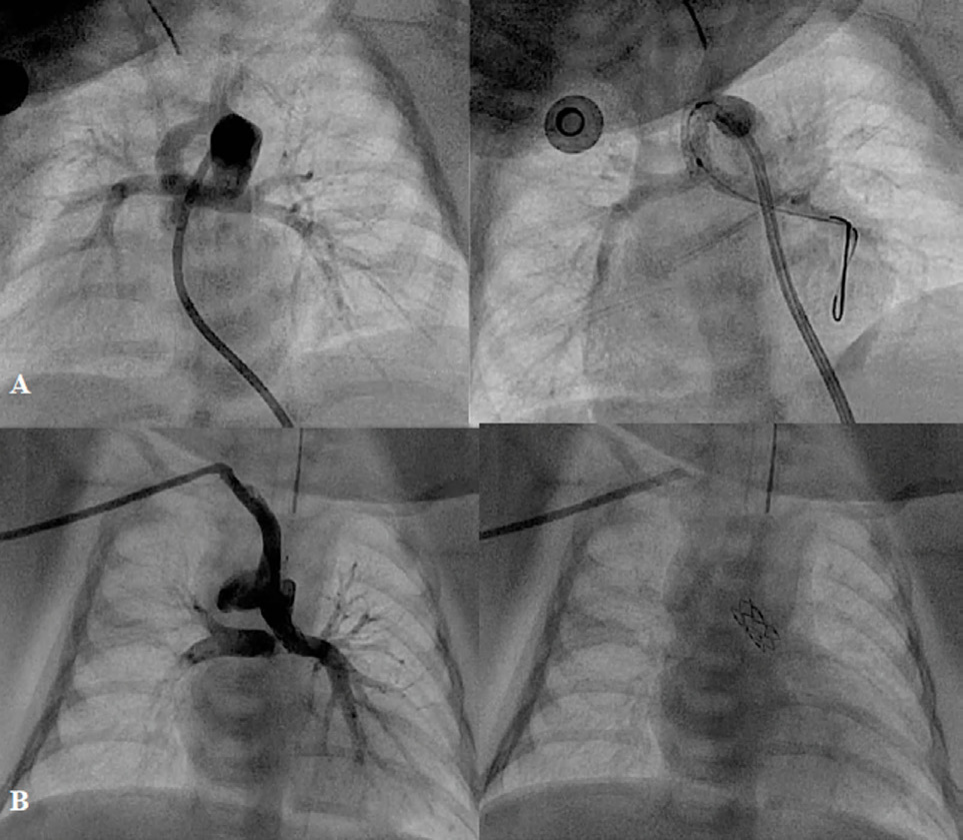
**Angiographic pictures of PDA stenting from different approaches: (A) from femoral artery and (B) from right axillary artery**.

Surgical systemic-to-pulmonary shunt (SPS), which mimicked the function of a PDA, has provided effective palliative treatment for duct-dependent pulmonary circulation defects for almost 70 years. In the recent years, SPS has been more commonly performed in patients with more complex defects, such as single-ventricle anatomy and pulmonary atresia than tetralogy of Fallot (TOF). In spite of wide clinical experience, neonatal mortality after SPS has ranged from 5 to 9% in the last two decades (47, 48). Recently, from the United Kingdom national database, the early mortality rate was 9.8%. In addition, at 1.5 years, 13.9% of patients had died, and 17.8% needed shunt reintervention (49). This is probably a reflection of the more complex types of defects in whom SPS has been performed. Currently, with the availability of flexible and low profile coronary artery stents, duct stenting can be performed safely through a 4F–5F sheath, as an alternative to SPS (Figure 4). However, not all the ducts can be stented. The success rate of PDA stenting depends on the complexity of the ductal morphology. Pulmonary atresia with intact ventricular septum is usually associated with a better chance of success with PDA stenting than the univentricular physiology or complex pulmonary atresia, in which the ductus arteriosus is usually tortuous (50, 51). Alwi and colleagues (52) reported an early mortality of 5.4% after ductal stenting with freedom from reintervention of 89% at 6 months and 55% at 12 months. It is reasonable to assume that none of the surgical and transcatheter palliative treatments for duct-dependent pulmonary circulation are perfect procedures. Both approaches have their own benefits and drawbacks, which require particular choice of the treatment in accordance with the particular defects, the availability of the devices, and the preference of the individual institution.

## Right Ventricular Outflow Tract Stenting

**Figure 5:**
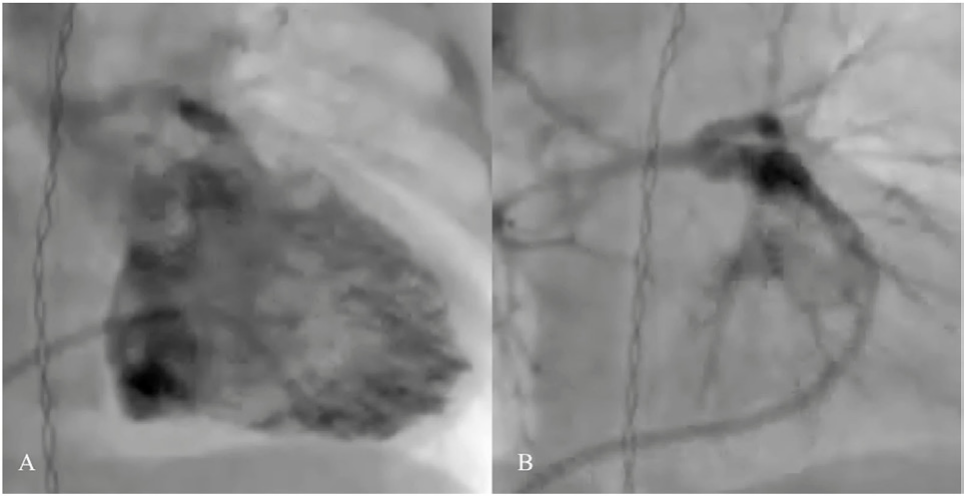
**Pre- (A) and post-procedural (B) angiography of the right ventricular outflow tract (RVOT) stenting in neonatal tetralogy of Fallot with severe infundibular stenosis**.

Right ventricular outflow tract (RVOT) stenting has been an effective palliative therapy in hypercyanotic neonates and young infants with TOF, in whom surgical therapy is considered unsuitable or a high risk (Figure 5). Stenting may promote symmetrical growth of the pulmonary arteries without branch distortion as shown from the literatures of significant increase of pulmonary branch diameters (53–55). Also, in those babies in whom the obstruction is predominately at infundibular level and the pulmonary valve is well functioning, stenting of the RVOT with pulmonary valve sparing placement of the stent may improve the oxygen saturation without compromising of the right ventricular outflow performance and without causing free pulmonary regurgitation. Some months later, once the patient is ready for a definitive repair, the RVOT stent can be removed with limited technical surgical difficulties. However, a majority of the RVOT stented patients may require transannular patch because of the size of the pulmonary valve annulus (56).

## Hybrid Palliation for Hypoplastic Left Heart Syndrome and Complex Single Ventricle

**Figure 6:**
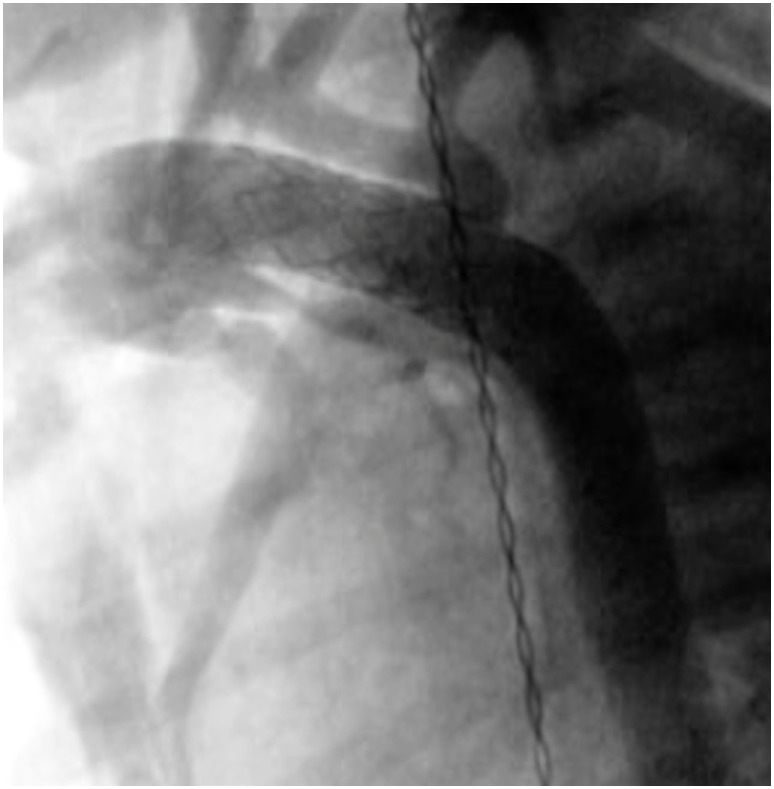
**Angiography after the ductus arteriosus stenting in neonate with aortic valve atresia and hypoplastic aortic arch**.

A combination of surgical and interventional catheterization as a targeted strategy, for example, by palliating a newborn with hypoplastic left heart syndrome (HLHS) using percutaneous ductal stenting (Figure 6) and bilateral branch pulmonary artery banding, was first described in 1992 (57). The aim of this approach is to minimize complications of HLHS Norwood Stage I operation for high-risk patients (e.g., small patient size, severe ventricular dysfunction, severe tricuspid regurgitation, small ascending aorta size, and multisystem organ failure) or as a bridge to transplantation. This strategy can also be performed in a variety of univentricular defects, in which systemic outflow or aortic arch appears hypoplastic (58, 59). Retrograde aortic arch obstruction with the PDA fully open is considered a contraindication to the hybrid stage I palliation, since the struts of the implanted stent may acutely compromise retrograde coronary blood flow (22). Reintervention after hybrid palliation is relatively frequent and reduction of mortality remains an opportunity to improve. Recently, Murphy et al. reported overall survival of 56.1% at a median follow-up of 32 months after hybrid palliation (59). Yerebakan et al. reported the follow-up results at 4.6 years after Giessen hybrid stage I palliation showing the operative mortality of hybrid stage I of 2.5%, comprehensive stage II of 4.9%, Fontan completion of 0%, and cumulative interstage mortality of 14.2% (60). The hybrid approach requires a novel mindset of collaboration between surgeons and interventionists along with high-end hybrid surgical suite. Hybrid palliation is a strong foundation of further collaboration for treatments of other complex congenital and structural heart defects.

## Catheter Interventions in Fontan Circulation

**Figure 7:**
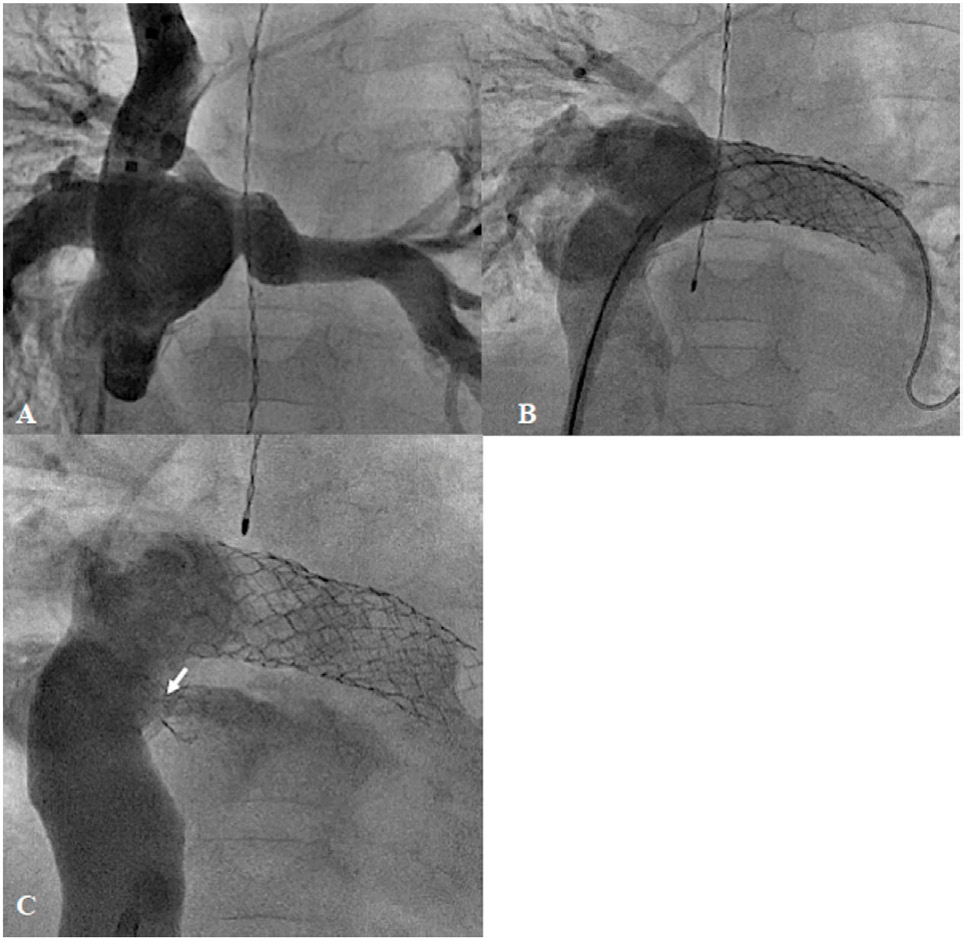
**Failing extra-cardiac Fontan circuit (A) treated by stenting of the hypoplastic left pulmonary artery (B) and diablo stenting of the restrictive fenestration (arrow) (C)**.

Surgical total cavopulmonary connection (TCPC), for the treatment of tricuspid atresia, was first described in 1971 by Fontan et al. (61). At present, with several modifications of the surgical technique, TCPC is the definitive treatment for most univentricular hearts. However, catheter intervention has become a major contributor pre-operatively or once these patients develop unstable hemodynamics, due to excessive pulmonary blood flow, low cardiac output, high venous pressure, progressive cyanosis, or obstruction in the pulmonary pathways. Pre-operative embolization of the excessive systemic-to-pulmonary collateral reduces the possibility of pulmonary hemorrhage and increased pulmonary arterial pressure due to overperfused lungs. Balloon angioplasty (BA) with or without stent implantation helps to create unobstructed pathways, which aim to optimize the pressures in this circuit (Figures 7A,B). Dilation or creation of a fenestration may stabilize the cardiac output in the early postoperative period (Figure 7C). In patients in whom cyanosis has persisted, occlusion of abnormal venous collaterals or previously created fenestration will improve systemic saturation. Completion of the Fontan or Glenn type of circulation may also be achieved percutaneously in carefully selected patients (62, 63). In addition, taking down the Fontan by enlargement of the fenestration and occlusion of the conduit to pulmonary artery has been reported in patients with early failure of the Fontan circulation (64).

Stenosis of the valves or vessels can be treated percutaneously. However, surgery remains treatment of choice in small children, with complex anatomy, or with residual lesions, or those in whom complications may have occurred after an intervention.

## Pulmonary Valvoplasty

**Figure 8:**
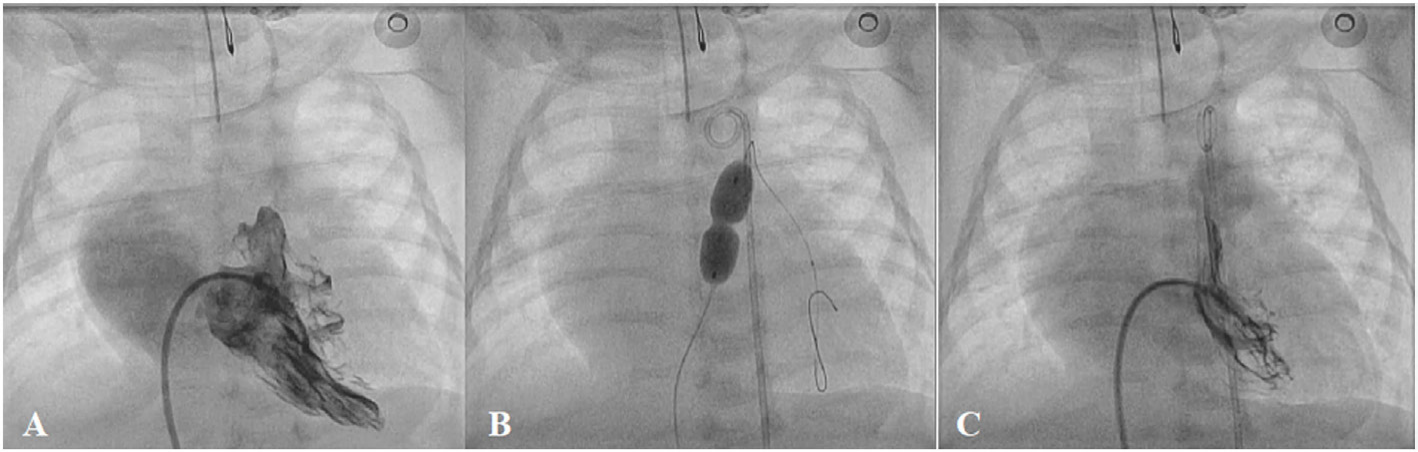
**Pre- (A), peri- (B), and post-procedural (C) angiography of the balloon pulmonary valvoplasty in neonatal critical pulmonary valve stenosis**.

Percutaneous balloon valvoplasty is feasible in most pulmonary valve stenosis patients (Figure 8). Post-procedural residual pressure gradient is usually <30 mmHg in 68–92% of the patients with low incidence of major adverse events after the procedure (65, 66). Approximately 80% of the patients are free from reintervention over 10 years (67, 68). Pulmonary valve insufficiency developed in 10–40% after balloon valvoplasty (69). This incidence and the severity appear to be less than after surgery (70). Surgical valvotomy, however, is the mainstay of treatment in patients with mainly subvalvar, supravalvar, and multi-level of obstructions.

## Aortic Valvoplasty

**Figure 9:**
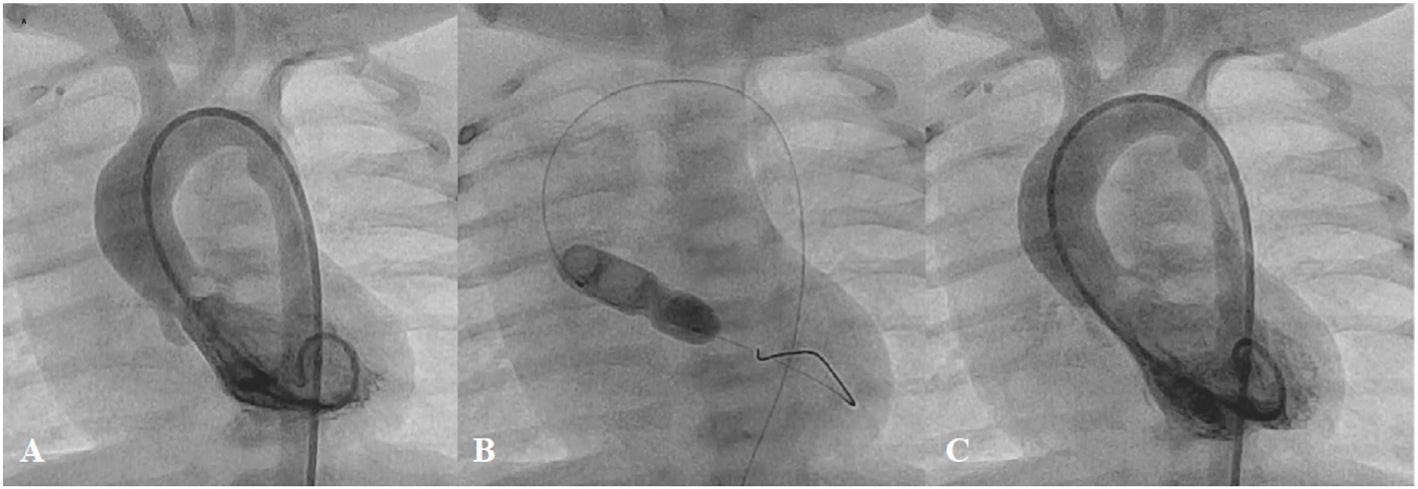
**Pre- (A), peri- (B), and post-procedural (C) angiography of the balloon aortic valvoplasty in neonate with severe aortic valve stenosis**.

Balloon aortic valvoplasty is now considered a safe procedure although it is less predictable with regard to the outcomes compared with balloon pulmonary valvoplasty. From the most recent multicentre registries (29, 70), 61.5–71% of procedures achieved a residual gradient ≤35 mmHg after single-balloon aortic valvoplasty. Moderate-to-severe aortic regurgitation appeared in 17.4–19% after treatment, while unplanned cardiac surgery was needed in 1%. Neonatal balloon aortic valvoplasty and mixed obstructions are considered to be at high risk for complications, death, and suboptimal outcomes (70) (Figure 9). Surgical correction is indicated in patients who develop major complications, such as severe aortic regurgitation, or aortic cusp avulsion, or those who have a residual significant pressure gradient, or multi-level of obstruction, or when associated with other cardiac lesions.

## Treatment of Coarctation of the Aorta

**Figure 10:**
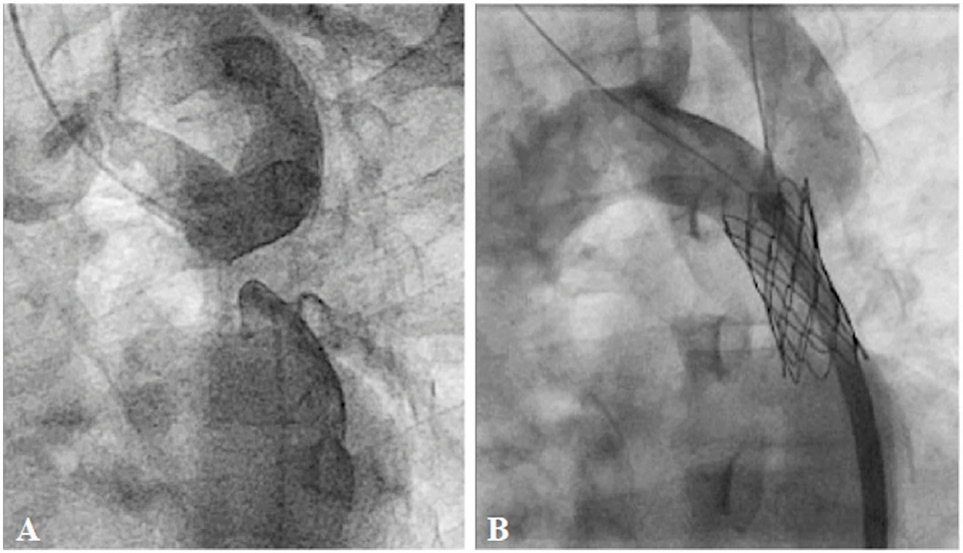
**Pre- (A) and post-procedural (B) angiography of the covered CP stent implantation in the interrupted descending aorta: Courtesy of Nageswara Rao Konati**.

Surgery is accepted as an effective and preferred treatment in neonates and infants with coarctation of the aorta (COA) (71, 72). However, in older children or adults, surgical morbidity is more frequent and may be detrimental (73, 74). BA has become an alternative treatment to surgery since 1983 (75, 76). Although the immediate results were relatively good, this procedure carries the risk of restenosis and aortic wall complications, especially after BA in native COA patients (77–79). Currently, BA is recommended as the treatment of choice only for children with aortic re-coarctation (29). Stent technology has evolved rapidly over the past two decades. Stent implantation is associated with effective outcomes in older children or adults with native or recurrent coarctation compared with surgery (74, 80–82). With open-cell stent design, complex coarctations can be treated safely without sacrificing the head and neck branches. Covered stents may be used to overcome or even prevent complications, such as aortic wall injury (83). With evolution of device technology, stent implantation in complex anatomy, such as nearly or completely interrupted arch, or multi-level obstruction, is feasible with favorable outcomes (84) (Figure 10). However, stent therapy in infants and small children with native COA has remained challenging for many years because of the need to expand the implanted stent to an adult size. Several breakable, over-dilatable, and biodegradable stents have been developed to overcome these limitations. Currently, stenting of native COA, even in small children, is feasible with excellent short-term outcomes (85–87).

## Stenosis of the Pulmonary Artery

**Figure 11:**
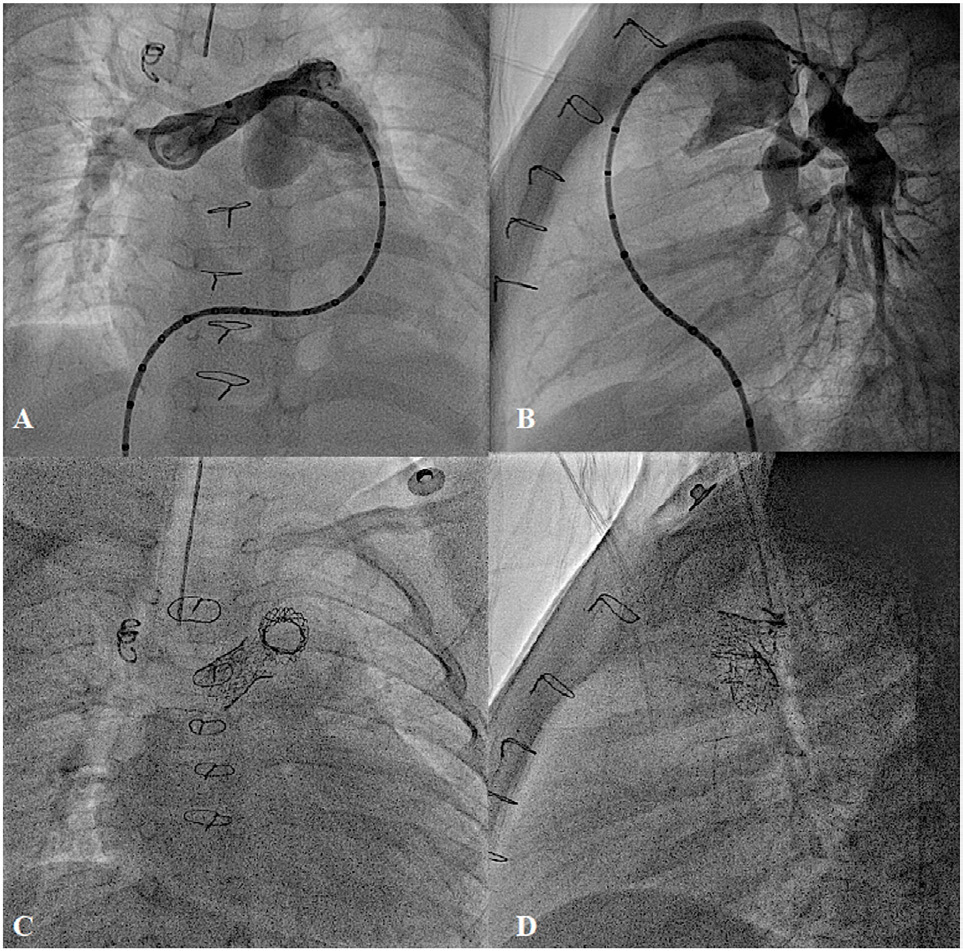
**Bilateral pulmonary artery stenting in post Rastelli type operation for type I truncus arteriosus with severe bilateral proximal pulmonary branches stenosis**. **(A,B)** Pre-procedure and **(C,D)** Post-stent implantation.

Stenosis of the proximal pulmonary artery branches, which are proximal to the hilum of the lungs, can either be tackled by surgery or catheter intervention. However, stenosis within the lung parenchyma can only be treated by endovascular approach, usually during catheterization or, less often, during hybrid surgery (88). Recurrent stenosis after surgery may occur in 35–40% of patients due to scar formation, distortion of the reconstructed vessels, or external compression (89, 90). In small children or patients with complex anatomy, primary BA may be used in severe main or branch pulmonary artery stenosis (22). However, in branch pulmonary artery stenoses, standard BA may have success rates of 50–60% with a recurrence rate of 15% and complication rate of 6–12% (88, 90–92). High-pressure and cutting balloons improved the success rates, especially in younger patients with resistant lesions (93, 94). However, with continuous improvement of the stent technology, in most patients, stenting has become a treatment of choice for branch pulmonary artery stenosis. Stents can be implanted effectively in patients with surgery-related stenosis, bifurcation stenosis, multi-level obstruction, or stenosis from the pressure effect of the adjacent structures (22, 95) (Figure 11). In growing children, however, they will require further dilation until the vessels reach adult diameter. In recurrent or complex stenoses, they usually need several attempts for pulmonary artery branch rehabilitation.

## Percutaneous Pulmonary Valve Implantation

**Figure 12:**
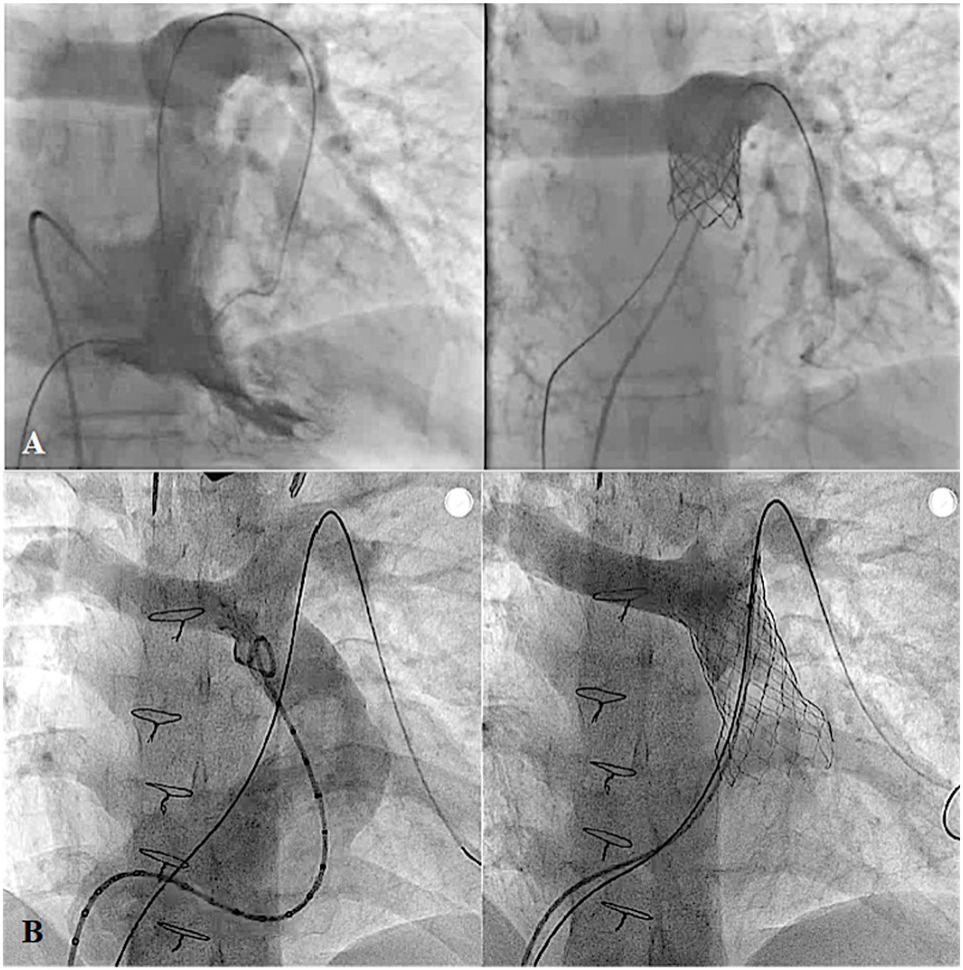
**Pre- and post-procedural angiography of the Melody valve implantation in post right ventricular outflow tract patch repaired pulmonary atresia with intact ventricular septum with severe pulmonary insufficiency (A) and Venus P-valve implantation in post transannular patch repaired tetralogy of Fallot with severe pulmonary regurgitation (B)**.

In order to accomplish complete bi-ventricular repair in various CHDs, such as tetralogy of Fallot, double outlet right ventricle, truncus arteriosus, or transposition of the great artery with ventricular septal defect, RVOT reconstruction with or without conduit replacement has been the mainstay of surgical treatment for decades. However, most of these reconstructed RVOTs will eventually develop dysfunction, and multiple surgical reinterventions may be needed during the lifetime of the patients (96, 97). Currently, percutaneous pulmonary valve implantation (PPVI) is considered an effective alternative treatment for conduit dysfunction (97) (Figure 12A). However, with limitations of the available valve diameters and the size of delivery systems, current exclusions of PPVI are dysfunctional large native RVOTs and body weight <30 kg (22, 97). To overcome the RVOT limitation, modifying the percutaneous valve into a self-expandable platform has shown feasibility to implant these valves into large native RVOTs up to 32 mm in diameter (15) (Figure 12B). In the near future, with continuous improvement of the valve design, PPVI will become a standard treatment in most dysfunctional post-surgical RVOTs. However, longevity of these valves is yet to be determined.

## Conclusion

Although interventional cardiologists have been able to treat many congenital heart defects without surgery, there are still numerous defects which need the surgeons. Furthermore, there is an increasing trend toward collaboration involving both the interventionist and the surgeons, with the aim of improving the patient outcomes.

## Author Contributions

WP: drafting and writing the work. SQ: revising it critically for important intellectual content; final approval of the version to be published. They both agree to be accountable for all aspects of the work in ensuring that questions related to the accuracy or integrity of any part of the work are appropriately investigated and resolved.

## Conflict of Interest Statement

The authors declare that the research was conducted in the absence of any commercial or financial relationships that could be construed as a potential conflict of interest.

## References

[1] Rubio-Alvarez V Limon-Larson R Soni J Valvulotomias intracardiacas por medio de un cateter. Arch Inst Cardiol Mexico (1953) 23:183–92. 13066260

[2] Kan SJ White RI Jr Mitchell SE Gardner TJ Percutaneous balloon valvuloplasty: a new method for treating congenital pulmonary valve stenosis. N Engl J Med (1982) 307:540–2. 10.1056/NEJM198208263070907 7099226

[3] McCrindle B Kan SJ. Long-term results after balloon pulmonary valvuloplasty. Circulation (1991) 83:1915–22. 10.1161/01.CIR.83.6.1915 2040044

[4] Rashkind WJ Miller WW Creation of an atrial septal defect without thoracotomy: a palliative approach to complete transposition of the great arteries. JAMA (1966) 196:991–2. 10.1001/jama.196.11.991 4160716

[5] Porstmann W Wierny L Warnke H Der Verschluss des Ductus arteriosus persistens ohne Thorakotomie (vorläufige Mitteilung). Thoraxchirurgie (1967) 15:199–203. 10.1055/s-0028-1100618 5239283

[6] King TD Mills NL Nonoperative closure of atrial septal defects. Surgery (1974) 75:383–8. 4811334

[7] Palmaz JC Richter GM Noeldge G Schatz RA Robison PD Gardiner GA Jr Intraluminal stents in atherosclerotic iliac artery stenosis: preliminary report of a multicenter study. Radiology (1988) 168:727–31. 10.1148/radiology.168.3.2970098 2970098

[8] Rees CR Palmaz JC Becker GJ Ehrman KO Richter GM Noeldge G Palmaz stent in atherosclerotic stenoses involving the ostia of the renal arteries: preliminary report of a multicenter study. Radiology (1991) 181:507–14. 10.1148/radiology.181.2.1924796 1924796

[9] O’Laughlin MP Perry SB Lock JE Mullins CE Use of endovascular stents in congenital heart disease. Circulation (1991) 83:1923–39. 10.1161/01.CIR.83.6.1923 2040045

[10] Qureshi SA Rosenthal E Tynan M Anjos R Baker EJ Transcatheter laser-assisted balloon pulmonary valve dilation in pulmonary valve atresia. Am J Cardiol (1991) 67:428–31. 10.1016/0002-9149(91)90056-Q 1994669

[11] Masura J Gavora P Formanek A Hijazi ZM. Transcatheter closure of secundum atrial septal defects using the new self-centering Amplatzer septal occluder: initial human experience. Cathet Cardiovasc Diagn (1997) 42:388–93. 10.1002/(SICI)1097-0304(199712)42:4<388::AID-CCD7>3.0.CO;2-7 9408617

[12] Masura J Walsh KP Thanopoulous B Chan C Bass J Goussous Y Catheter closure of moderate- to large-sized patent ductus arteriosus using the new Amplatzer duct occluder: immediate and short-term results. J Am Coll Cardiol (1998) 31:878–82. 10.1016/S0735-1097(98)00013-8 9525563

[13] Thanopoulos BD Tsaousis GS Konstadopoulou GN Zarayelyan AG. Transcatheter closure of muscular ventricular septal defects with the Amplatzer ventricular septal defect occluder: initial clinical applications in children. J Am Coll Cardiol (1999) 33:1395–9. 10.1016/S0735-1097(99)00011-X 10193744

[14] Grube E Laborde JC Gerckens U Felderhoff T Sauren B Buellesfeld L Percutaneous implantation of the corevalve self-expanding valve prosthesis in high-risk patients with aortic valve disease: the Siegburg first-in-man study. Circulation (2006) 114:1616–24. 10.1161/CIRCULATIONAHA.106.639450 17015786

[15] Promphan W Prachasilchai P Siripornpitak S Qureshi SA Layangool T. Percutaneous pulmonary valve implantation with the venus P-valve: clinical experience and early results. Cardiol Young (2015) 19:1–13. 10.1017/S1047951115001067 26088820

[16] Finan E Mak W Bismilla Z McNamara PJ. Early discontinuation of intravenous prostaglandin E1 after balloon atrial septostomy is associated with an increased risk of rebound hypoxemia. J Perinatol (2008) 28:341–6. 10.1038/jp.2008.11 18337745

[17] Beattie LM McLeod KA. Prostaglandin E_2_ after septostomy for simple transposition. Pediatr Cardiol (2009) 30:447–51. 10.1007/s00246-008-9357-2 19083139

[18] Hiremath G Natarajan G Math D Aggarwal S. Impact of balloon atrial septostomy in neonates with transposition of great arteries. J Perinatol (2011) 31:494–9. 10.1038/jp.2010.196 21273986

[19] Cinteza E Carminati M Balloon atrial septostomy – almost half a century after. Maedica (Buchar) (2013) 8:280–4. 24371500 PMC3869120

[20] Wang K Pan X Tang Q Pang Y. Catheterization therapy vs surgical closure in pediatric patients with patent ductus arteriosus: a meta-analysis. Clin Cardiol (2014) 37:188–94. 10.1002/clc.22238 24395607 PMC6649407

[21] Lam JY Lopushinsky SR Ma IW Dicke F Brindle ME. Treatment options for pediatric patent ductus arteriosus: systematic review and meta-analysis. Chest (2015) 148:784–93. 10.1378/chest.14-2997 25835756

[22] Feltes TF Bacha E Beekman RH III Cheatham JP Feinstein JA Gomes AS Indications for cardiac catheterization and intervention in pediatric cardiac disease: a scientific statement from the American Heart Association. Circulation (2011) 123:2607–52. 10.1161/CIR.0b013e31821b1f10 21536996

[23] Ghasemi A Pandya S Reddy SV Turner DR Du W Navabi MA Trans-catheter closure of patent ductus arteriosus-what is the best device? Catheter Cardiovasc Interv (2010) 76:687–95. 10.1002/ccd.22393 20815044

[24] Brunetti MA Ringel R Owada C Coulson J Jennings JM Hoyer MH Percutaneous closure of patent ductus arteriosus: a multiinstitutional registry comparing multiple devices. Catheter Cardiovasc Interv (2010) 76:696–702. 10.1002/ccd.22538 20690153

[25] Rohit MK Gupta A Transcatheter closure of large patent ductus arteriosus using custom made devices. Cathet Cardiovasc Interv (2014). 10.1002/ccd.25349 24323820

[26] Francis E Singhi AK Lakshmivenkateshaiah S Kumar RK. Transcatheter occlusion of patent ductus arteriosus in pre-term infants. JACC Cardiovasc Interv (2010) 3:550–5. 10.1016/j.jcin.2010.01.016 20488412

[27] Kenny D Morgan GJ Bentham JR Wilson N Martin R Tometzki A Early clinical experience with a modified Amplatzer ductal occluder for transcatheter arterial duct occlusion in infants and small children. Catheter Casdiovasc Interv (2013) 82:526–33. 10.1002/ccd.24522 22718329

[28] Abu Hazeem AA Gillespie MJ Thun H Munson D Schwartz MC Dori Y Percutaneous closure of patent ductus arteriosus in small infants with significant lung disease may offer faster recovery of respiratory function when compared to surgical ligation. Catheter Cardiovasc Interv (2013) 82:526–33. 10.1002/ccd.25032 23723091

[29] Moore JW Vincent RN Beekman RH III Benson L Bergersen L Holzer R Procedural results and safety of common interventional procedures in congenital heart disease: initial report from the National Cardiovascular Data Registry. J Am Coll Cardiol (2014) 64:2439–51. 10.1016/j.jacc.2014.09.045 25500227

[30] Little DC Pratt TC Blalock SE Krauss DR Cooney DR Custer MD. Patent ductus arteriosus in micropreemies and full-term infants: the relative merits of surgical ligation versus indomethacin treatment. J Pediatr Surg (2003) 38:492–6. 10.1053/jpsu.2003.50086 12632374

[31] Philip R Rush Waller B III Agrawal V Wright D Arevalo A Zurakowski D Morphologic characterization of the patent ductus arteriosus in the premature infant and the choice of transcatheter occlusion device. Catheter Cardiovasc Interv (2016) 87:310–7. 10.1002/ccd.26287 26525611

[32] Suchon E Pieculewicz M Tracz W Przewlocki T Sadowski J Podolec P. Transcatheter closure as an alternative and equivalent method to the surgical treatment of atrial septal defect in adults: comparison of early and late results. Med Sci Monit (2009) 15:CR612–7. 19946231

[33] Kaya MG Baykan A Dogan A Inanc T Gunebakmaz O Dogdu O Intermediate-term effects of transcatheter secundum atrial septal defect closure on cardiac remodeling in children and adults. Pediatr Cardiol (2010) 31:474–82. 10.1007/s00246-009-9623-y 20084376

[34] Knepp MD Rocchini AP Lloyd TR Aiyagari RM. Long-term follow up of secundum atrial septal defect closure with the Amplatzer septal occluder. Congenit Heart Dis (2010) 5:32–7. 10.1111/j.1747-0803.2009.00358.x 20136855

[35] Sarris GE Kirvassilis G Zavaropoulos P Belli E Berggren H Carrel T Surgery for complications of trans-catheter closure of atrial septal defects: a multi-institutional study from the European Congenital Heart Surgeons Association. Eur J Cardiothorac Surg (2010) 37:1285–90. 10.1016/j.ejcts.2009.12.021 20353896

[36] Roberts WT Parmar J Rajathurai T. Very late erosion of Amplatzer septal occluder device presenting as pericardial pain and effusion 8 years after placement. Catheter Cardiovasc Interv (2013) 82:E592–4. 10.1002/ccd.24755 23172721

[37] Abaci A Unlu S Alsancak Y Kaya U Sezenoz B. Short and long term complications of device closure of atrial septal defect and patent foramen ovale: meta-analysis of 28,142 patients from 203 studies. Catheter Cardiovasc Interv (2013) 82:1123–38. 10.1002/ccd.24875 23412921

[38] Menting ME Cuypers JA Opić P Utens EM Witsenburg M van den Bosch AE The unnatural history of the ventricular septal defect: outcome up to 40 years after surgical closure. J Am Coll Cardiol (2015) 65:1941–51. 10.1016/j.jacc.2015.02.055 25953746

[39] Holzer R de Giovenni J Walsh KP Tometzki A Goh T Hakim F Transcatheter closure of perimembranous ventricular septal defects using the Amplatzer membranous VSD occluder: immediate and midterm results of an international registry. Catheter Cardiovasc Interv (2006) 68:620–8. 10.1002/ccd.20659 16969878

[40] Fu YC Bass J Amin Z Radtke W Cheatham JP Hellenbrand WE Transcatheter closure of perimembranous ventricular septal defects using the new Amplatzer membranous VSD occluder: results of the U.S. phase I trial. J Am Coll Cardiol (2006) 47:319–25. 10.1016/j.jacc.2005.09.028 16412854

[41] Chungsomprasong P Durongpisitkul K Vijarnsorn C Soongswang J Lê TP. The results of transcatheter closure of VSD using Amplatzer^®^ device and Nit Occlud^®^ Lê coil. Catheter Cardiovasc Interv (2011) 78:1032–40. 10.1002/ccd.23084 21648053

[42] Lee SM Song JY Choi JY Lee SY Paik JS Chang SI Transcatheter closure of perimembranous ventricular septal defect using Amplatzer ductal occluder. Catheter Cardiovasc Interv (2013) 82:1141–6. 10.1002/ccd.24810 23554093

[43] Yang J Yang L Yu S Liu J Zuo J Chen W Transcatheter versus surgical closure of perimembranous ventricular septal defects in children: a randomized controlled trial. J Am Coll Cardiol (2014) 63:1159–68. 10.1016/j.jacc.2014.01.008 24509270

[44] Bai Y Xu XD Li CY Zhu JQ Wu H Chen SP Complete atrioventricular block after percutaneous device closure of perimembranous ventricular septal defect: a single-center experience on 1046 cases. Heart Rhythm (2015) 12:2132–40. 10.1016/j.hrthm.2015.05.014 25981147

[45] Wang S Zhuang Z Zhang H Zhen J Lu Y Liu J Perventricular closure of perimembranous ventricular septal defects using the concentric occluder device. Pediatr Cardiol (2014) 35:580–6. 10.1007/s00246-013-0823-0 24196912

[46] Zhang S Zhu D An Q Tang H Li D Lin K. Minimally invasive perventricular device closure of doubly committed sub-arterial ventricular septal defects: single center long-term follow-up results. J Cardiothorac Surg (2015) 15:119. 10.1186/s13019-015-0326-6 26374555 PMC4572622

[47] Tamisier D Vouhé PR Vernant F Lecá F Massot C Neveux JY. Modified Blalock-Taussig shunts: results in infants less than 3 months of age. Ann Thorac Surg (1990) 49:797–801. 10.1016/0003-4975(90)90026-3 1692681

[48] Dirks V Prêtre R Knirsch W Valsangiacomo Buechel ER Seifert B Schweiger M Modified Blalock Taussig shunt: a not-so-simple palliative procedure. Eur J Cardiothorac Surg (2013) 44:1096–102. 10.1093/ejcts/ezt172 23539419

[49] Dorobantu DM Pandey R Sharabaini MT Mahani AS Angelini GD Martin RP Indications and results of systemic to pulmonary shunts: results from a national database†. Eur J Cardiothorac Surg (2016) 49(6):1553–63. 10.1093/ejcts/ezv435 26768397

[50] Udink Ten Cate FE Sreeram N Hamza H Agha H Rosenthal E Qureshi SA. Stenting the arterial duct in neonates and infants with congenital heart disease and duct-dependent pulmonary blood flow: a multicenter experience of an evolving therapy over 18 years. Catheter Cardiovasc Interv (2013) 82:E233–43. 10.1002/ccd.24878 23420699

[51] Sivakumar K Bhagyavathy A Coelho R Satish R Krishnan P. Longevity of neonatal ductal stenting for congenital heart diseases with duct-dependent pulmonary circulation. Congenit Heart Dis (2012) 7:526–33. 10.1111/j.1747-0803.2012.00657.x 22548982

[52] Alwi M Choo KK Latiff HA Kandavello G Samion H Mulyadi MD. Initial results and medium-term follow-up of stent implantation of patent ductus arteriosus in duct-dependent pulmonary circulation. J Am Coll Cardiol (2004) 44:438–45. 10.1016/j.jacc.2004.03.066 15261945

[53] Stumper O Ramchandani B Noonan P Mehta C Bhole V Reinhardt Z Stenting of the right ventricular outflow tract. Heart (2013) 99:1603–8. 10.1136/heartjnl-2013-304155 23846613

[54] Castleberry CD Gudausky TM Berger S Tweddell JS Pelech AN. Stenting of the right ventricular outflow tract in the high-risk infant with cyanotic teratology of Fallot. Pediatr Cardiol (2014) 35:423–30. 10.1007/s00246-013-0796-z 24096718

[55] Bertram H Emmel M Ewert P Grohmann J Haas NA Jux C Stenting of native right ventricular outflow tract obstructions in symptomatic infants. J Interv Cardiol (2015) 28:279–87. 10.1111/joic.12198 25990981

[56] Barron DJ Ramchandani B Murala J Stumper O De Giovanni JV Jones TJ Surgery following primary right ventricular outflow tract stenting for Fallot’s tetralogy and variants: rehabilitation of small pulmonary arteries. Eur J Cardiothorac Surg (2013) 44:656–62. 10.1093/ejcts/ezt188 23650024

[57] Gibbs JL Rothman MT Rees MR Parsons JM Blackburn ME Ruiz CE. Stenting of the arterial duct: a new approach to palliation for pulmonary atresia. Br Heart J (1992) 67:240–5. 10.1136/hrt.67.3.240 1372815 PMC1024799

[58] Taqatqa A Diab KA Stuart C Fogg L Ilbawi M Awad S Extended application of the hybrid procedure in neonates with left-sided obstructive lesions in an evolving cardiac program. Pediatr Cardiol (2015) 3:465–71. 10.1007/s00246-015-1301-7 26538212

[59] Murphy MO Bellsham-Revell H Morgan GJ Krasemann T Rosenthal E Qureshi SA Hybrid procedure for neonates with hypoplastic left heart syndrome at high-risk for norwood: midterm outcomes. Ann Thorac Surg (2015) 100:2286–92. 10.1016/j.athoracsur.2015.06.098 26433522

[60] Yerebakan C Valeske K Elmontaser H Yörüker U Mueller M Thul J Hybrid therapy for hypoplastic left heart syndrome: myth, alternative, or standard? J Thorac Cardiovasc Surg (2016) 151(4):1112–23.e5. 10.1016/j.jtcvs.2015.10.066 26704055

[61] Fontan F Mounicot F Baudet F Simmoneau J Gordo J Gouffrant J Correction de I’atrsie tricuspididienne: report de deux cas “carriqes” par 1 utilisation d’une technique chirqicale nouvelle. Ann Chir Thorac Cardiovasc (1971) 10:39–47. 5101717

[62] McMahon CJ el-Said HG Mullins CE. Transcatheter creation of an atriopulmonary communication in the Hemi-Fontan or Glenn circulation. Cardiol Young (2002) 12:196–9. 10.1017/S1047951102000422 12018731

[63] Galantowicz M Cheatham JP Lessons learned from the development of a new hybrid strategy for the management of hypoplastic left heart syndrome. Pediatr Cardiol (2005) 26:190–9. 10.1007/s00246-004-0962-4 16179977

[64] Hallbergson A Mascio CE Rome JJ. Transcatheter Fontan takedown. Catheter Cardiovasc Interv (2015) 86:849–54. 10.1002/ccd.25963 25945427

[65] Mendelsohn AM Banerjee A Meyer RA Schwartz DC. Predictors of successful pulmonary balloon valvuloplasty: 10-year experience. Cathet Cardiovasc Diagn (1996) 39:236–43. 10.1002/(SICI)1097-0304(199611)39:3<236::AID-CCD6>3.0.CO;2-F 8933964

[66] Holzer RJ Gauvreau K Kreutzer J Trucco SM Torres A Shahanavaz S Safety and efficacy of balloon pulmonary valvuloplasty: a multicenter experience. Catheter Cardiovasc Interv (2012) 80:663–72. 10.1002/ccd.23473 22422728

[67] Garty Y Veldtman G Lee K Benson L. Late outcomes after pulmonary valve balloon dilatation in neonates, infants and children. J Invasive Cardiol (2005) 17:318–22. 16003007

[68] Rao PS. Percutaneous balloon pulmonary valvuloplasty: state of the art. Catheter Cardiovasc Interv (2007) 69:747–63. 10.1002/ccd.20982 17330270

[69] O’Connor BK Beekman RH Lindauer A Rocchini A. Intermediate-term outcome after pulmonary balloon valvuloplasty: comparison with a matched surgical control group. J Am Coll Cardiol (1992) 20:169–73. 10.1016/0735-1097(92)90154-F 1607519

[70] Torres A Vincent JA Everett A Lim S Foerster SR Marshall AC Balloon valvuloplasty for congenital aortic stenosis: multi-center safety and efficacy outcome assessment. Catheter Cardiovasc Interv (2015) 86:808–20. 10.1002/ccd.25969 26032565

[71] Wright GE Nowak CA Goldberg CS Ohye RG Bove EL Rocchini AP. Extended resection and end-to-end anastomosis for aortic coarctation in infants: results of a tailored surgical approach. Ann Thorac Surg (2005) 80:1453–9. 10.1016/j.athoracsur.2005.04.002 16181886

[72] Burch PT Cowley CG Holubkov R Null D Lambert LM Kouretas PC Coarctation repair in neonates and young infants: is small size or low weight still a risk factor? J Thorac Cardiovasc Surg (2009) 138(3):547–52. 10.1016/j.jtcvs.2009.04.046 19698833

[73] Choudhary P Canniffe C Jackson DJ Tanous D Walsh K Celermajer DS. Late outcomes in adults with coarctation of the aorta. Heart (2015) 101:1190–5. 10.1136/heartjnl-2014-307035 25810155

[74] Yeaw X Murdoch DJ Wijesekera V Sedgwick JF Whight CM Pohlner PG Comparison of surgical repair and percutaneous stent implantation for native coarctation of the aorta in patients ≥15years of age. Int J Cardiol (2016) 203:629–31. 10.1016/j.ijcard.2015.11.052 26580346

[75] Sperling DR Dorsey TJ Rowen M Gazzaniga AB. Percutaneous transluminal angioplasty of congenital coarctation of the aorta. Am J Cardiol (1983) 51:562–4. 10.1016/S0002-9149(83)80097-6 6218748

[76] Lock JE Bass JL Amplatz K Fuhrman BP Castaneda-Zuniga W Balloon dilation angioplasty of aortic coarctations in infants and children. Circulation (1983) 68:109–16. 10.1161/01.CIR.68.1.109 6221828

[77] Tynan M Finley JP Fontes V Hess J Kan J. Balloon angioplasty for the treatment of native coarctation: results of valvuloplasty and angioplasty of congenital anomalies registry. Am J Cardiol (1990) 65:790–2. 10.1016/0002-9149(90)91389-N 2316462

[78] Mendelsohn AM Lloyd TR Crowley DC Sandhu SK Kocis KC Beekman RH. Late follow-up of balloon angioplasty in children with a native coarctation of the aorta. Am J Cardiol (1994) 74:696–700. 10.1016/0002-9149(94)90312-3 7942528

[79] Fawzy ME Fathala A Osman A Badr A Mostafa MA Mohamed G Twenty-two years of follow-up results of balloon angioplasty for discreet native coarctation of the aorta in adolescents and adults. Am Heart J (2008) 156:910–7. 10.1016/j.ahj.2008.06.037 19061706

[80] Carr JA. The results of catheter-based therapy compared with surgical repair of adult aortic coarctation. J Am Coll Cardiol (2006) 21(47):1101–7. 10.1016/j.jacc.2005.10.063 16545637

[81] Forbes TJ Garekar S Amin Z Zahn EM Nykanen D Moore P Procedural results and acute complications in stenting native and recurrent coarctation of the aorta in patients over 4 years of age: a multi-institutional study. Catheter Cardiovasc Interv (2007) 70:276–85. 10.1002/ccd.21164 17630670

[82] Meadows J Minahan M McElhinney DB McEnaney K Ringel R COAST Investigators*. Intermediate outcomes in the prospective, multicenter coarctation of the aorta stent trial (COAST). Circulation (2015) 131:1656–64. 10.1161/CIRCULATIONAHA.114.013937 25869198

[83] Tretter JT Jones TK McElhinney DB. Aortic wall injury related to endovascular therapy for aortic coarctation. Circ Cardiovasc Interv (2015) 8(9):e002840. 10.1161/CIRCINTERVENTIONS.115.002840 26291468

[84] Suárez de Lezo J Romero M Pan M Suárez de Lezo J Segura J Ojeda S Stent repair for complex coarctation of aorta. JACC Cardiovasc Interv (2015) 8:1368–79. 10.1016/j.jcin.2015.05.018 26315741

[85] Schranz D Zartner P Michel-Behnke I Akintürk H. Bioabsorbable metal stents for percutaneous treatment of critical recoarctation of the aorta in a newborn. Catheter Cardiovasc Interv (2006) 67:671–3. 10.1002/ccd.20756 16575923

[86] Quandt D Ramchandani B Bhole V Penford G Mehta C Dhillon R Initial experience with the cook formula balloon expandable stent in congenital heart disease. Catheter Cardiovasc Interv (2015) 85(2):259–66. 10.1002/ccd.25543 24824198

[87] Grohmann J Sigler M Siepe M Stiller B. A new breakable stent for recoarctation in early infancy: preliminary clinical experience. Catheter Cardiovasc Interv (2016) 87(4):E143–50. 10.1002/ccd.26393 26724789

[88] Kan J Marvin W Bass J Muster A Murphy J Balloon angioplasty branch pulmonary artery stenosis: results of the valvuloplasty and angioplasty congenital anomalies registry. Am J Cardiol (1990) 65:798–801. 10.1016/0002-9149(90)91391-I 2316463

[89] Luhmer I Ziemer G. Coarctation of the pulmonary artery in neonates. Prevalence, diagnosis, and surgical treatment. J Thorac Cardiovasc Surg (1993) 106(5):889–94. 8231213

[90] Trant CA Jr O’Laughlin MP Ungerleider RM Garson A Jr. Cost-effectiveness analysis of stents, balloon angioplasty, and surgery for the treatment of branch pulmonary artery stenosis. Pediatr Cardiol (1997) 18:339–44. 10.1007/s002469900195 9270100

[91] Hoshina M Tomita H Kimura K Ono Y Yagihara T Echigo S. Factors determining peripheral pulmonary artery stenosis remodeling in children after percutaneous transluminal balloon angioplasty. Circ J (2002) 66:345–8. 10.1253/circj.66.345 11954947

[92] Gentles T Lock J Perry S. High pressure balloon angioplasty for branch pulmonary artery stenosis: early experience. J Am Coll Cardiol (1993) 22:867–72. 10.1016/0735-1097(93)90205-F 8354826

[93] Bergersen L Gauvreau K Justino H Nugent A Rome J Kreutzer J Randomized trial of cutting balloon compared with high-pressure angioplasty for the treatment of resistant pulmonary artery stenosis. Circulation (2011) 124:2388–96. 10.1161/CIRCULATIONAHA.111.018200 22042887

[94] Ing FF Khan A Kobayashi D Hagler DJ Forbes TJ. Pulmonary artery stents in the recent era: immediate and intermediate follow-up. Catheter Cardiovasc Interv (2014) 84:1123–30. 10.1002/ccd.25567 24910458

[95] Niemantsverdriet MB Ottenkamp J Gauvreau K Del Nido PJ Hazenkamp MG Jenkins JK. Determinants of right ventricular outflow tract conduit longevity: a multinational analysis. Congenit Heart Dis (2008) 3:176–84. 10.1111/j.1747-0803.2008.00190.x 18557880

[96] de Ruijter FT Weenink I Hitchcock FJ Meijboom EJ Bennink GB. Right ventricular dysfunction and pulmonary valve replacement after correction of tetralogy of Fallot. Ann Thorac Surg (2002) 73:1794–800. 10.1016/S0003-4975(02)03586-5 12078771

[97] Holzer RJ Hijazi ZM. Transcatheter pulmonary valve replacement: state of the art. Catheter Cardiovasc Interv (2016) 87:117–28. 10.1002/ccd.26263 26423185

